# Evaluation of 10-Year Selection for Virus Resistance in a Mass Breeding Program

**DOI:** 10.3390/insects17020137

**Published:** 2026-01-24

**Authors:** Emma Bossuyt, Marleen Brunain, Lina De Smet, Ellen Danneels, Dirk C. de Graaf

**Affiliations:** 1 Honeybee Valley, Department of Biochemistry and Microbiology, Faculty of Sciences, Ghent University, Krijgslaan 287, S33, 9000 Ghent, Belgium; ellen.danneels@ugent.be (E.D.); dirk.degraaf@ugent.be (D.C.d.G.); 2 Laboratory of Molecular Entomology and Honeybee Pathology (L-MEB), Department of Biochemistry and Microbiology, Faculty of Sciences, Ghent University, Krijgslaan 287, S2, 9000 Ghent, Belgium; marleen.brunain@ugent.be (M.B.); lina.desmet@ugent.be (L.D.S.)

**Keywords:** SOV, viruses, honey bee, selection, mass breeding

## Abstract

Viral infections pose a major threat to honey bee health. While viruses are typically controlled indirectly through efforts of attaining Varroa resistance, the heritable trait suppressed *in ovo* virus infection (SOV) provides a direct avenue for selecting virus resistance. This study evaluated the potential of this trait using data collected within an established mass breeding selection program. Drone egg samples collected from honey bee colonies in Flanders (2015–2024) were screened for four viruses to determine the queen’s SOV status. Queens are classified as SOV-positive if no viral particles are detected in their sample, and as SOV-negative if genomic material from at least one of these viruses is present. The proportion of SOV-positive queens significantly increased over time, regardless of maternal background, and targeted breeding from SOV-positive maternal lines enhanced the likelihood of producing SOV-positive offspring. Simultaneously, the prevalence and viral load values of several viruses decreased over time. These findings demonstrate that selective breeding for SOV-positivity can improve virus resistance in managed honey bee populations. There is even a potential to raise the SOV trait occurrence by incorporating targeted mating within selection programs. Therefore, future research should focus on the combined selection for SOV through targeted breeding and mating, alongside Varroa-resistant traits.

## Simple Summary

Honey bees continue to face health threats from viral infections. Most virus-control efforts focus on reducing Varroa mite infestations, which spread viruses within honey bee colonies. However, some honey bee queens possess the heritable trait suppressed *in ovo* virus infection, which means these queens naturally produce drone eggs free from honey bee viruses. This provides the opportunity to directly select for virus resistance. In this study, we used data collected within an ongoing mass breeding selection program (2015–2024) in Flanders. This data includes drone egg samples collected from bee colonies and analyzed for four honey bee viruses. Hereby, we evaluate the incorporation of this trait within a selection program. Our results show that the number of virus-free egg-producing queens increased significantly over time. Breeding from these queens further increased the likelihood that their offspring would also produce virus-free eggs. In parallel, both the presence and intensity of several virus infections decreased over time. These findings show that targeted breeding, together with targeted mating, for virus-free egg-producing queens can successfully improve virus resistance in managed honey bee populations and that, in the future, this trait should be implemented together with Varroa-resistant traits in selection programs.

## 1. Introduction

The deterioration of honey bees has received significant scientific attention due to the pressing threat to the viability of beekeeping. An inevitable enemy of managed honey bees remains the Varroa mite, *Varroa destructor*. The ectoparasite’s reproductive cycle is synchronized to the host’s development in brood cells, feeding on both adult bees and larvae, more specifically by draining their hemolymph and fat body tissue [1]. Eventually, this leads to individual bee damage such as reduced flight performance and/or a shorter lifespan [2,3]. Concomitantly, viruses can be transferred because *V. destructor* is a vector for various honey bee viruses. Worldwide, more than 20 bee viruses are known [4], with deformed wing virus (DWV), black queen cell virus (BQCV), acute bee paralysis virus (ABPV), chronic bee paralysis virus (CBPV) and sacbrood virus (SBV) being the most common ones, each with its typical symptoms [5]. Since the arrival of the Varroa mite, the incidence of these viruses is substantially elevated, with often multiple viruses being present in one colony [5,6]. Once a honey bee colony is contaminated, further transmission, within or between colonies, can occur vertically and horizontally. In horizontal transmission, viruses circulate between individuals of the same generation. Horizontal intra-colony transmission is the result of direct body contact, trophallaxis, larval food or cannibalism of virus-infected pupae. Horizontal inter-colony transmission can occur due to foraging (common flower visits), robbing or drifting. Vertical transmission refers to the spread of viruses from parents to their offspring via infected eggs or contaminated drone semen (intra-colony) or swarming (inter-colony) [4,7,8,9]. Although horizontal transmission may seem the most efficient method due to the high number of bees within a colony, it was shown that vertical transmission also occurs rather frequently [6].

Direct suppression of viruses by the honey bee is challenging; however, bees possess different defense mechanisms against pathogen transmission, such as propolis (an antibacterial substance to line the colony walls and seal the nest cavity), antibiotic activity in pollen and honey stores or removing/cleaning dead, potentially diseased, carcasses from the hive [8]. Indirect suppression of viruses is achievable by lowering the amount of mites in the hives. Multiple efforts are performed by the beekeeper to restrain mite populations in the hives. The initial response to a Varroa infestation was chemical treatment, because this was deemed the best option against colony collapse. In most cases, honey bee colonies would collapse within 2 to 3 years if they are left without periodic treatment [10]. However, repetitive application of such chemicals can pollute bee products as well as harm the bees; moreover, mites eventually develop resistance to these products. Alternatives to the chemical approach are biotechnical techniques (e.g., drone trapping). Although these techniques have gained interest in recent years, their actual use remains scarce. A more desirable outcome would be for honey bees to be capable of defending themselves against parasites without direct human intervention. Certain traits enable honey bees to protect themselves against *V. destructor,* such as Varroa-sensitive hygiene (VSH), (auto- and allo-) grooming, suppression of the mite’s reproductive success [10,11]. More resistance traits limit the survival of *V. destructor* within a colony. Ideally, these (closely) resistance-linked traits are heritable and can be easily implemented in honey bee selection by beekeepers [12].

Another heritable trait that can be selected for, regarding resistance against viruses, is ‘suppressed *in ovo* virus infection’ (SOV), described as the absence of viruses in 10 pooled drone eggs [7,13]. The foundation for this novel trait lies in a previous study by Ravoet et al. [6], in which vertical virus transmission was measured non-destructively by examining worker eggs of Belgian breeding queens for commonly occurring bee viruses. The results of these sanitary screenings showed high infection rates. Therefore, de Graaf et al. [7] continued these yearly sanitary controls on drone eggs. These samples are unfertilized and thus carry only the alleles of the queen and are ideal to estimate the heritability of the virus status. Although the underlying mechanism behind the SOV trait is not yet known, it was demonstrated that the virus status of a drone egg can be partially explained by the queen’s genetics and thus represents a heritable trait [7]. Furthermore, the study of Claeys Bouuaert et al. [13] concluded that the SOV trait achieves increased virus resistance expressed in the descending honey bee queens. Hence, it was suggested that the SOV trait be implemented in a queen breeding program, as it allows direct selection for virus resistance.

In Flanders, we (Honeybee Valley at Ghent University) launched a selection breeding program in 2016, with its first active testing season in 2017, aiming to improve the resilience of honey bee colonies while maintaining productivity and ease of handling. We gave support by defining and standardizing different tests for defensive, productivity and behavioral traits, executing some laboratory tests, such as determining the virus status of drone eggs for the SOV trait, centralizing data collection using a website application (Breed It), processing the data following a mass-breeding protocol (=ranking) and providing advice at the individual queen (/beekeeper) level. The SOV trait is used as an exclusion criterion in this mass-selection program: only if a queen scores good for resilience and there are no viruses found in her sampled drone eggs (SOV+ queen), we advise our beekeepers to breed from this queen. In addition to this targeted breeding advice, we give targeted mating advice for the newly bred virgin queen. Ideally, a new queen bred from an SOV+ maternal line should be mated with an SOV+ drone line. This can be achieved using instrumental insemination (II) or the land-mating station Kreverhille, where drone lines with a known SOV+ status are positioned.

Since 2015, the SOV trait has been determined yearly. Therefore, in this study, we aimed to evaluate the use of this trait in a mass breeding selection program after many years of selective breeding, and the following questions were put forward. First, how has participation in SOV testing evolved over the past decade? To what extent have our efforts led to an improvement of the colonies’ resilience in terms of the occurrence of the SOV trait and general virus load of the eggs? To what extent has targeted breeding via the maternal and/or targeted mating via the paternal line determined the occurrence of this trait? Additionally, due to the participating queens’ background data being available, we were also able to assess if the bee race or the type of mating plays a role in the occurrence of this trait.

## 2. Materials and Methods

### 2.1. Sample Collection

From 2015 to 2024, Flemish beekeepers, participating in the selection program of Honeybee Valley, sampled a self-selected number of colonies at their apiary, preferably with a defined genetic and management background, from different regions in Flanders, Belgium, which share broadly comparable climatic conditions. Across the study period, the number of SOV-tested queens per beekeeper typically ranged between 3 and 8, with a mean of ≈5 SOV-tested queens per beekeeper. No strict additional inclusion criteria were imposed, but queen testing was conducted within the standardized framework of the selection program (see Supplementary Materials Figures S1 and S2). This allowed for a comparable number of SOV-tested queens every sampling year. In 2015, the starting year of SOV sampling, the number of SOV-tested queens was lower in comparison to the other sampling years. See Supplementary Materials Table S1, for extra information regarding the number of participating beekeepers and the arithmetic mean number of SOV-tested queens per beekeeper for every sampling year. Participants collected 10 drone eggs per colony in a pre-labeled 1.5 mL tube between April and June, to check for the presence of the SOV trait. To avoid contamination, beekeepers used a different toothpick for every colony. After collection, the eggs were immediately stored at −20 °C. After transportation to the laboratory (maintaining the cold chain), the samples were stored at −80 °C until virus status determination.

### 2.2. Virus Screening in Eggs

As this work constitutes an assessment of long-term data rather than a methodological study, the full protocol is not reiterated here; instead, it is provided in the cited references. All egg samples were processed via RNA extraction (using the RNeasy Lipid tissue mini kit (“Qiagen, Hilden, Germany”)) and cDNA synthesis, and then screened for the presence of four viruses using RT-PCR or RT-qPCR, following the protocols described by de Graaf et al. [7] and the follow-up improvements mentioned in Claeys Bouuaert et al. [14]. From 2015 until 2019, DWV(A), SBV, ABPV and BQCV were screened using RT-PCR. From 2020 to 2024, DWV(complex), SBV, ABPV and BQCV were screened using RT-qPCR. The viral copies for the different viruses were determined by absolute quantification, and the integrity of the RNA was analyzed using actin as target gene.

### 2.3. Statistics

Data cleaning and analysis were conducted using RStudio version 4.4.2 (RStudio, Boston, MA, USA). Visualization was conducted in both RStudio and Microsoft Excel (Version 251 (Build 16.0.19426.20218)). To improve data compliance with statistical analysis, viral loads were natural-log (ln) transformed and for data visualization, viral loads were log10-transformed. For proportion/count SOV data and number of viruses per infected queen data, generalized linear mixed models (GLMM) (Poisson or binomial distribution), which include fixed effects parameters (sampling year/generation) and random effects (beekeeper), were used to analyze trends over sampling years and generations. Where no random effect was included, the equivalent generalized linear model (GLM) (binomial distribution) was applied. Associations between SOV status and explanatory variables (e.g., winter survival and honey bee subspecies) were assessed using chi-squared tests of independence. For the probability of a sample having virus genome copies (i.e., virus-positive sample) (presence/absence), mixed logistic regression models (GLMM) (binomial distribution) were applied, with sampling year (treated as a continuous predictor) as a fixed effect and beekeeper as a random intercept. Viral load values of virus-positive samples were modeled using log-normal mixed models (Gaussian distribution) with the same fixed and random structure. If the log-normal model failed to converge or had a clearly worse fit, a Gamma GLMM with a log link function was fitted as an alternative. For the effect of the mating type and honey bee subspecies, linear mixed models were used. Models were checked (using residual diagnostics, dispersion, AIC, etc.) to ensure they met the necessary assumptions. Where relevant, results are reported as model coefficients with standard errors and *p*-values; for interpretation, model coefficients are reported on the link/log scale, and their exponentiated values are presented on the response scale (odds ratios for binomial models; multiplicative changes for Poisson/log-normal/Gamma models). All models and final inferences explicitly note limitations due to sample size and the number and distribution of sampling years.

## 3. Results

### 3.1. Descriptive Statistics

From 2015 to 2024, the virus status of 1921 egg samples (further referred to as “queens”) originating from 98 Flemish queen breeders was determined. The dataset contained samples of 1697 *A. mellifera carnica* queens, 120 *A. mellifera* Buckfast queens, 24 *A. mellifera mellifera* queens and 80 queens of an unknown species. The drone egg samples were analyzed for DWV, ABPV, BQCV and SBV and were scored 0 or 1 (0 = absence; 1 = presence) for each of the viruses. For DWV, the threshold value for virus positivity was set at 10^6^ virus genome copies per 1 egg. This threshold was selected following concordance testing between RT-PCR and RT-qPCR (See Supplementary Materials Table S2). For all other viruses, no threshold value was used. The infection rate ranges from zero to four viruses per queen. Over the years, 1388 queens were virus-free for the screened viruses, 431 queens were infected with a single virus, 78 queens with two viruses present, 21 queens with three viruses present and three queens with four viruses present. In the span of 10 years, 59 queens tested positive for ABPV, 234 for BQCV, 231 for DWV (DWV(A) or DWV(complex) and 138 for SBV. The SOV trait is negative (SOV−) if the total number of viruses ranges from one to four. If all four viruses are absent in the sample, the SOV trait is positive (SOV+). In total, 1388 queens were SOV+ and 533 queens were SOV−. Overall, 125 queens were subjected to targeted mating (i.e., mating with an SOV+ drone line) and 1796 queens were subjected to non-targeted mating. Winter survival of the sampled colonies was recorded for the winter following each sampling year, since 2021.

### 3.2. Participation in SOV Testing

In 2015, only 83 queens were involved in the sanitary screenings. However, by the second year, the number of SOV-tested queens increased to 195, and by the last year, 2024, this number had risen to 220 (Table 1, B). Overall, the data suggested that the number of SOV-tested queens remained relatively stable across sampling years. To assess the temporal trend of the number of SOV-tested queens over sampling years, a GLMM with beekeeper included as a random effect was fitted. The Poisson GLMM exhibited signs of overdispersion; therefore, a negative binomial GLMM was employed. The model estimated a small positive effect of sampling year on the number of SOV-tested queens (β = 0.019 ± 0.013 SE, 95% CI: –0.006 to 0.045), but this effect was not statistically significant (*p* = 0.13). Thus, no clear trend was detected.

Although SOV testing was already performed in 2015 and 2016, the testing season within the selective breeding program started in 2017, at which point SOV testing became an integral part of the selection process. In the following analysis, the observed proportions refer to the share of queens tested for SOV relative to all queens participating in the breeding program (Table 1, A–B). In the initial years of the breeding program (2017–2018), nearly all queens in the selection group were tested for their SOV status (≈96%), but by 2020, participation had declined to ≈67%. Since then, participation has fluctuated but has never exceeded ≈ 80% (Table 1, C). To assess whether participation changed systematically over time (2017–2024), a binomial generalized linear model (GLM), with sampling year as the predictor, was fitted. The data suggested a gradual decline. The model confirmed a decline in the proportion of SOV testing (β = −0.22 ± 0.026 SE, *p* < 2 × 10^−16^), corresponding to an estimated 20% decrease per year (95% CI: 0.76 to 0.84). Thus, there is a decline in participation for SOV testing across sampling years.

### 3.3. Improvement of the SOV+ Status

Table 1 (D–E) shows the occurrence of the SOV+ status regardless of the SOV status of the previous generations. Overall, there was a gradual increase in the number of SOV+ queens from 2017 to 2019, followed by a temporary decrease in 2020. This decrease coincided with the transition from RT-PCR to the more sensitive RT-qPCR, which may reflect a methodological effect rather than a biological decline. Thereafter, the number of SOV+ queens increased again from 2020 to 2022. Here, the data suggested a slight increase in the observed number of SOV+ queens across sampling years (2015–2024). To analyze the total number of SOV+ queens over time, a GLMM with a negative binomial distribution, including sampling year as a fixed effect and beekeeper as a random intercept to account for repeated measures, was fitted. The model showed adequate dispersion and provided the best variance fit. The effect of year was positive and statistically significant (β = 0.073 ± 0.017 SE, *p* = 2.83 × 10^−5^), indicating that the number of SOV+ queens increased over time, corresponding with an estimated increase of ≈8% per year (95% CI: 1.04–1.11). Thus, there is strong evidence for an upward trend in the number of SOV+ queens between 2015 and 2024.

Examining the observed percentage of SOV+ queens each sampling year reveals a similar trend to the total number of SOV+ queens. There is a clear increase from 2017 to 2019, followed by a sharp decline in 2020, and then a subsequent increase from 2020 to 2022. Initially, only ≈57% of tested queens were SOV+, rising to ≈ 85% by 2024 (Table 1, E). To evaluate whether the proportion of SOV+ queens changed over time, we fitted a GLMM with a negative binomial distribution, including year as a fixed effect and beekeeper as a random intercept to account for repeated measures. The model showed adequate fit and captured the variance structure better than alternatives. The sampling year had a significant positive effect on the proportion of SOV+ queens (β = 0.071 ± 0.012 SE, *p* < 8.21 × 10^−9^), corresponding to an approximate 7% increase per year (95% CI: 1.05–1.10). Thus, there is strong evidence that the proportion of SOV+ queens increased steadily between 2015 and 2024.

Extra analyses were performed to assess the association between a queen’s SOV status and their winter survival, and between a queen’s SOV status and her honey bee subspecies. The chi-squared tests indicated that SOV status was not associated with winter survival (χ^2^ = 0.04, df = 1, *p* = 0.84). Similarly, no significant relationship was observed between SOV status and honey bee subspecies (χ^2^ = 6.97, df = 4, *p* = 0.14).

Within the selection group, multiple generations of queens were sometimes tested in subsequent years. The longest chain of consecutive tested generations is six; however, there were only five queens in this group, and this group was therefore negligible. Examining the number of SOV+ queens per generation, regardless of the SOV status of the maternal line, the most abundant group of consecutive generations consists of two generations, with 284 SOV+ queens (Table 2, A). The percentage of SOV+ queens, compared to all SOV-tested, appears to increase slightly with each generation, starting at a high level of about ≈ 74% in the first generation (Table 2, B). A binomial GLMM showed that generation had a positive effect on the proportion of SOV+ queens (β = 0.13 ± 0.077 SE, *p* = 0.081), corresponding to an approximate 14% increase in the odds of a queen being SOV+ per generation (95% CI: 0.98–1.33). Given the limited number of generations (*n* = 5) and the non-significance, these results should be interpreted cautiously and are presented primarily to illustrate the observed trend.

### 3.4. Improvement of the SOV+ Trait Due to Targeted Breeding

**Figure 1:**
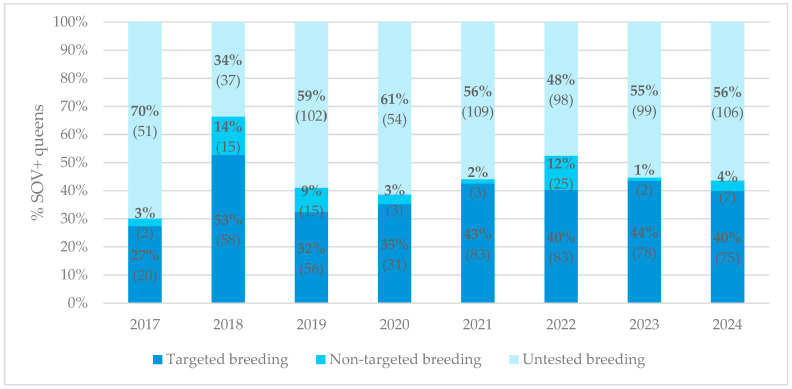
Distribution of the percentage of SOV+ queens per sampling year (2017–2024) with different phenotypic maternal lines (targeted, non-targeted and untested breeding). Values in parentheses indicate the absolute number of queens.

Since setting up SOV testing within the breeding program in 2017, our participants have been advised to breed only from SOV+ maternal lines. Figure 1 illustrates the percentage and absolute number of SOV+ queens bred from SOV+ maternal lines (i.e., targeted breeding), from SOV− maternal lines (i.e., non-targeted breeding) or from untested maternal lines (i.e., untested breeding) per sampling year. In every sampling year, except for 2018, the largest percentage of SOV+ queens descended from maternal lines whose SOV status was unknown. Nevertheless, the absolute number of SOV+ queens originating from targeted breeding exceeded those from non-targeted breeding.

Examining the observed percentages of SOV+ queens, in 2017, the first year of the breeding program, only ≈27% of SOV+ queens were the result of targeted breeding. In 2018, when our breeding advice could be followed, the percentage of SOV+ queens increased notably. From 2019 onwards, this percentage increased, although it never exceeded ≈50% in later years (Table 1, G).

To evaluate whether the proportion of SOV+ queens due to targeted breeding indeed increased over time, a Poisson GLMM with sampling year as a fixed effect, beekeeper as a random intercept, was fitted. The model showed a good fit. The sampling year had a significant positive effect (β = 0.092 ± 0.025 SE, *p* = 0.0002), corresponding to an approximate increase of 10% per year in the proportion of SOV+ queens (95% CI: 1.04–1.15). Despite variation among beekeepers, these results indicate that the proportion of SOV+ queens due to targeted breeding increased steadily between 2017 and 2024.

**Figure 2:**
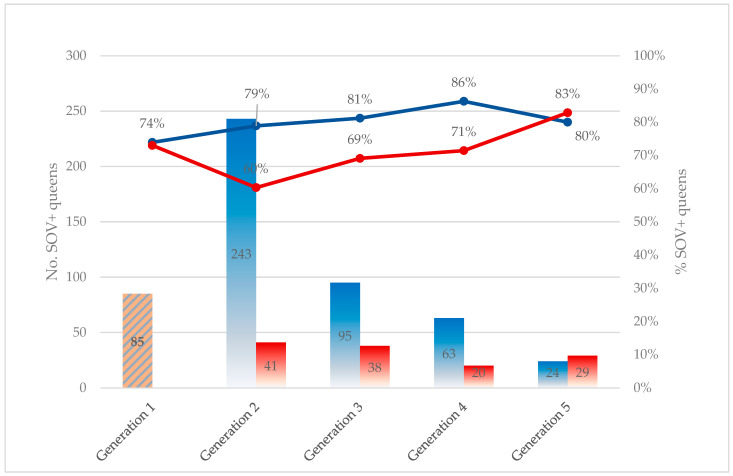
Evolution of SOV+ queens with different phenotypic maternal lines across generations (1–5). Left *y*-axis (bars): Distribution of the number of SOV+ queens in subsequent generations (1–5), distinguishing queens derived from consecutive targeted breeding in blue, and queens derived from consecutive non-targeted breeding in red, applied from generation 2 onward. Right *y*-axis (lines): For every generation (1–5), the percentage of SOV+ queens relative to all queens derived from consecutive targeted breeding in blue, or relative to all queens derived from consecutive non-targeted breeding in red.

Across generations, the percentage of SOV+ queens due to consecutive targeted breeding appeared to increase from generation 1 to generation 4, followed by a slight decrease in generation 5 (Figure 2, blue line). A binomial GLM showed that generation had a positive effect on the proportion of SOV+ queens (β = 0.17 ± 0.098 SE, *p* = 0.075), corresponding to an approximate 19% increase in the odds of a queen being SOV+ per generation (95% CI: 0.98–1.44). Given the limited number of generations (*n* = 5) and the non-significance, these results should be interpreted cautiously and are presented primarily to illustrate the observed trend. In comparison, for queens bred from an SOV− maternal line (non-targeted breeding), the percentage of SOV+ queens dropped sharply after the first non-targeted breeding, but then gradually increased again by the fifth generation of consecutive non-targeted breeding (Figure 2, red line).

### 3.5. Improvement of the SOV Trait Due to Targeted Breeding and Targeted Mating

To investigate the effect of targeted mating on the SOV status, information on the maternal line’s mating and its SOV status was required. However, the majority of maternal matings were unknown, and in most cases, they were not SOV tested. Because of these low numbers, data from different sampling years were combined. Of all known maternal matings, only ≈20% occurred via II or RKH, and among those, ≈41% of the drone lines used were SOV+ (i.e., targeted mating) (Table 1, H–I). Notably, the other ≈59% of used mating (drone) lines were not SOV tested, i.e., an SOV− mating line was never used. Of all known maternal lines, ≈54% were SOV tested, of which ≈ 86% were SOV+ (i.e., targeted breeding) (Table 1, J–K). The combination of targeted breeding and targeted mating was rare; however, in the 46 cases where it occurred (from 2017 to 2024), ≈83% of queens were SOV+ (Table 1, L–N).

### 3.6. Description of the SOV− Status

The SOV− status was further examined, which, in this study, indicates the presence of viral infections. First, the viral load values of ABPV, BQCV, DWV and SBV were assessed over sampling years. As qPCR analysis was introduced in 2020, the evaluation of temporal trends in virus infections was therefore limited to 2020 and later. Mixed logistic regressions (presence/absence) were modeled to determine the probability of virus detection. A log-normal mixed model was fitted to examine the viral load over sampling years. Both types of models had the beekeeper included as a random effect.

**Figure 3:**
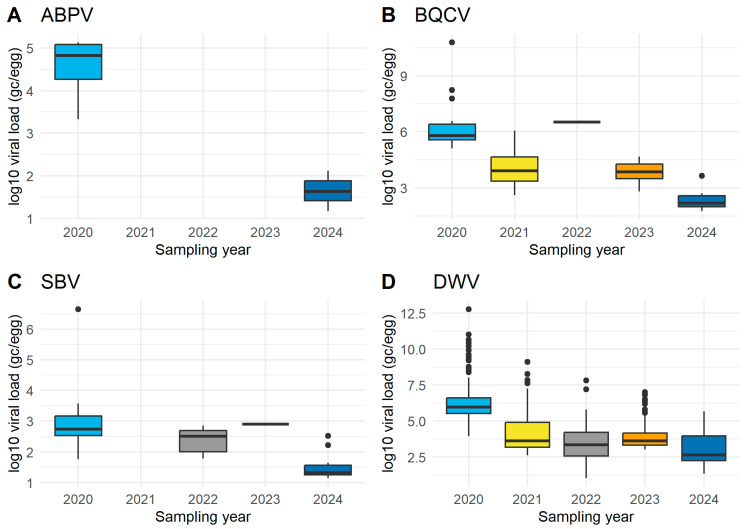
Boxplots with log_10_-transformed viral load (genome copies per egg) in egg samples per sampling year. (**A**) ABPV = acute bee paralysis virus, (**B**) BQCV = black queen cell virus, (**C**) SBV = sacbrood virus, and (**D**) DWV = deformed wing virus. Boxes show medians with interquartile ranges; whiskers represent the range excluding outliers (points).

The probability of detecting ABPV was higher in 2024 compared to 2020 (β = 0.99 ± 0.36 SE, *p* = 0.006). The model suggested that viral load was significantly lower in 2024 than in 2020 (β = –1.46 ± 0.20 SE, *p* = 9.15 × 10^−13^) (Figure 3A). This pattern indicates that while ABPV was detected more frequently in 2024, the viral loads in positive colonies were reduced compared to 2020. Given the limited number of positive samples and the restriction to two sampling years (Table 3, A; *n* = 14 from 10 beekeepers), these results should be considered preliminary and interpreted with caution.

BQCV was the most frequently detected virus over the five years, compared to the other viruses (Table 3, B; *n* = 111 from 43 beekeepers). The probability of detection declined significantly over time (β = –0.40 ± 0.09 SE, *p* = 1.20 × 10^−5^), indicating that later years had lower odds of a positive sample than earlier years. Next, restricting to BQCV-positive samples, the model showed a strong negative time effect: the BQCV viral load values declined over time (β = –1.50 ± 0.19 SE, *p* = 5.37 × 10^−15^) (Figure 3B). Together, these results suggest both decreasing frequency and decreasing intensity of BQCV infections over 5 sampling years.

For DWV, data were available across four sampling years (Table 3, C; *n* = 111 from 62 beekeepers). The probability of detecting DWV in colonies declined significantly over time (β = –0.66 ± 0.06 SE, *p* < 2 × 10^−16^). When restricting the analysis to only positive samples, the model revealed that DWV viral load values decreased significantly over years (β = –1.67 ± 0.11 SE, *p* < 2 × 10^−16^) (Figure 3D). Thus, DWV not only declined in detection probability but also in infection intensity across sampling years.

For SBV, data were available from four sampling years (Table 3, D; *n* = 44 from 20 beekeepers). Therefore, a weak negative effect of time on the probability of detection (β = –0.22 ± 0.11, *p* ≈ 0.05) was suggested. SBV viral load values decreased significantly over time (β = –1.13 ± 0.22 SE, *p* = 3.46 × 10^−7^) (Figure 3C). However, the limited number of positive samples and uneven distribution across years mean that these results should be interpreted with caution. In general, the overall viral load of the eggs has improved over the years.

Second, the effect of mating type and honey bee subspecies (each separately) on the viral load values was assessed. For all viruses, we fitted linear mixed-effects models with the log-transformed viral load as the response variable, the type of mating and the honey bee subspecies as fixed effects, and the beekeeper as a random effect to account for repeated sampling. In some models, certain factors could not be included because only a single level was represented in the data for that virus. In none of the models for the different viruses did honey bee subspecies or type of mating have a significant effect.

**Figure 4:**
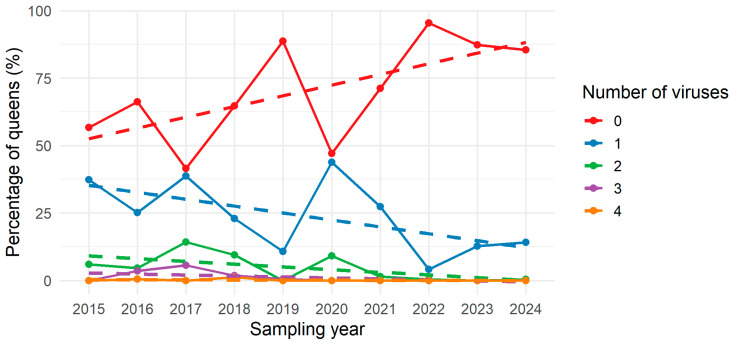
Percentage of queens (%) with different numbers of viruses (0–4) detected per egg sample across sampling years (2015–2024). Solid lines connect the observed percentages of queens for each sampling year, whereas dotted lines indicate trend lines fitted to the observed data.

As previously mentioned, the probability of queens being SOV+ (not infected with viruses) increased over time, or in other words, the probability of queens being SOV− (infected with 1–4 viruses) decreased across sampling years (2015–2024). Figure 4 illustrates that the number of viruses (1–4) detected per colony has declined in recent years compared to earlier years. For queens that were infected (≥1 virus), an attempt to model the number of viruses per queen across years using a negative binomial mixed model with beekeeper as a random effect was performed. However, the data did not contain sufficient variation to support a reliable model fit. As the last occurrence of four viruses in a single egg sample was observed in 2018 and the last occurrence of three viruses in 2019.

## 4. Discussion

Identifying viral infections within a colony can be challenging, as viruses often persist as covert, asymptomatic infections [15]. Typically, when symptoms become apparent, the viral load is considerably elevated, and the colony is already approaching collapse [15]. In contrast, the mite load in managed colonies is a more readily observable parameter. Historically, various mite-control strategies have been implemented within apicultural practices. By limiting mite infestations, the transmission of viruses is indirectly mitigated. Conversely, the suppressed *in ovo* virus infection (SOV) trait provides a direct method for controlling virus status by heritable means. Accordingly, this trait can be integrated into breeding programs as a selection tool for virus resistance.

With data on the SOV trait collected since 2015 and integrated into a breeding program from 2017 onwards, we are able to evaluate its progression. Within this selection program, the SOV status is a mandatory exclusion criterion, determined through non-invasive sampling of ten drone eggs per colony, collected by beekeepers and analyzed in the laboratory. Most of the virus screening results are published in [6,7,14].

### 4.1. Participation in SOV Testing

During the initial years of incorporating the SOV trait into the Flemish breeding program, participation rates were notably high (2017 & 2018: approximately 96%). The decline in participation observed in 2020 may be attributed to the COVID-19 pandemic. The fluctuations in participation over the past four years can be attributed to natural turnover within the selection group, with some beekeepers discontinuing their involvement while others newly joined the program. Although the overall proportion of participation for the SOV trait has decreased over time, consistently high participation rates (>65%) among beekeepers indicate that the SOV status remains a significant factor in their colony selection processes.

### 4.2. Improvement of the SOV+ Status

When evaluating improvements in the colonies’ resilience, specifically the occurrence of the SOV+ status, our results provide evidence of an overall increase in the occurrence of SOV+ queens between 2015 and 2024. Both the absolute number and the proportion of SOV+ queens showed a consistent upward trend over time, suggesting that the SOV+ trait is becoming increasingly prevalent. However, as these data include queens of various backgrounds, this trend cannot yet be attributed solely to selective breeding efforts.

The temporary decline in 2020 can largely be explained by a methodological change from RT-PCR to qPCR. The latter technique is substantially more sensitive, allowing for the detection of lower viral loads that would previously have remained undetected with RT-PCR (threshold of 10^8^ for SBV, 10^7^ for BQCV, and 10^6^ for ABPV) [14]. Therefore, the apparent decrease in SOV+ queens in 2020 should be interpreted as a methodological effect rather than a biological effect. Fluctuations in SOV+ occurrence in later years may also reflect changes in program participation. Each year, new beekeepers can join the program, while others may discontinue their involvement. New beekeepers often test queens without a prior selection history, which increases the likelihood of SOV− queens entering the dataset and may temporarily reduce the overall SOV+ proportion.

Several additional factors may influence the SOV status, and some plausible factors are considered in this discussion. Colonies across Flanders experience diverse management and environmental conditions, which may further contribute to variation among years and apiaries [16]. Ideally, more remote apiaries and good management (low-stress conditions) should be implemented to reduce viral infections [16]. However, in the Flemish breeding program, we do not impose any restrictions on beekeeping practices, enabling sampling of as many colonies as possible in Flanders. Although climate is sometimes proposed as a potential influencing factor, empirical evidence for its impact on viral infections in honey bee colonies remains limited. Among other findings, the study by Ramos-Cuellar et al. [17] determined the prevalence and intensity of viral infections in Mexican honey bee colonies in subtropical and temperate climates, but no significant effect of climate was observed for either DWV or BQCV infection prevalence and intensity. In this study, samples were collected from different regions within Flanders; however, these regions do not differ substantially in climatic conditions. Therefore, it is unlikely that climate contributed to the observed variation in the SOV status, resulting from variation in the virus occurrence. The timing of sample collection is another important consideration. Virus dynamics are known to fluctuate throughout the year [18]; evidently, the SOV status can change between seasons. This was demonstrated in the study of Claeys Bouuaert et al. [14], samples collected in the spring exhibited significantly higher DWV and BQCV infection frequencies than samples collected in the summer. Therefore, standardizing sampling time is crucial for reliable comparisons across years and studies. The data of the samples included in this study were collected within a consistent seasonal window (spring), minimizing seasonal bias.

The generational analysis provides a complementary perspective. Although based on a relatively limited dataset, the results indicate a slight, though non-significant, increase in the proportion of SOV+ queens with each subsequent generation. This observation suggests that the SOV+ trait may be maintained and potentially strengthened across generations, possibly through active selection or as a correlated response to management practices. The second generation represented the largest group, as multiple daughter queens were reared from a single mother queen. However, not all of these daughter queens were used for further breeding, which explains why the number of SOV-tested queens did not increase exponentially across generations.

### 4.3. Improvement of the SOV+ Trait Due to Targeted Breeding

An important question arising from the observed temporal increase in SOV+ queens is whether this improvement can be attributed to our efforts in targeted breeding practices. Selection programs implementing targeted breeding with managed honey bee populations demonstrate a faster and more controlled approach to improving the occurrence of certain resistance traits than natural selection, as, for example, shown for Varroa-Sensitive Hygiene (VSH) [19,20]. In this study, targeted breeding refers to breeding from SOV+ maternal lines, whereas non-targeted breeding refers to breeding from SOV− maternal lines. Since establishing the breeding program in 2017, participating beekeepers have been advised to breed exclusively from SOV+ queens. However, Figure 1 illustrates that this recommendation was not consistently followed. In 2017, most queens still originated from maternal lines of unknown SOV status, but by 2018, a larger proportion were bred from confirmed SOV+ queens. Across all sampling years, however, the majority of queens continued to originate from untested maternal lines. This pattern may reflect the annual influx of new participants who join the selection program without prior access to SOV-tested queens. Another plausible explanation is that beekeepers occasionally lose their SOV+ queens before they can rear new generations from them, forcing them to breed from queens with an unknown or SOV− status. Nevertheless, the overall proportion of SOV+ queens produced through targeted breeding was consistently higher than that achieved through non-targeted breeding. The statistical analyses confirm a gradual increase in the proportion of SOV+ queens produced through targeted breeding between 2017 and 2024. This finding demonstrates that breeding from SOV+ maternal lines enhances the likelihood of producing SOV+ offspring. Although winter mortality has only been monitored since 2021, this pattern is unlikely to result from differential survival between SOV+ and SOV− queens, as SOV status was not significantly associated with winter survival. Thus, the higher proportion of SOV+ queens in the targeted breeding group appears to result from deliberate breeding decisions rather than differential overwintering success. Additionally, no significant association between honey bee subspecies and SOV status was observed, suggesting that subspecies did not have a detectable effect in this dataset. However, the significantly increasing trend is relatively modest, suggesting that breeding exclusively through the maternal line may not fully account for the overall improvement in the SOV+ trait. Other contributing factors—such as drone influence (mating), environmental variation, nutritional availability or unintentional selection by management practices—may also influence the SOV status [21,22].

The generation-level analysis provides additional insight into the long-term effects of targeted breeding. The data indicated a gradual increase in the proportion of SOV+ queens across successive generations, rising from 74% in the first generation to 86% by the fourth generation, followed by a slight decline to 80% in the fifth generation. The small decrease observed in the fifth generation likely reflects the limited number of queens tested in later generations rather than a true biological decline. Although the observed positive trend did not reach statistical significance and is not strictly linear, it aligns with the hypothesis that targeted breeding could gradually favor the SOV+ status across successive generations.

Interestingly, the difference in the proportion of SOV+ queens between targeted and non-targeted breeding diminished with each subsequent generation, and by the fifth generation, the likelihood of producing an SOV+ queen was higher in the non-targeted group. This apparent convergence may be attributed to several factors. First, the number of queens available for multi-generational analysis was limited, particularly in later generations, thereby reducing statistical power and increasing uncertainty. Second, the heritability of the SOV trait is relatively moderate (h^2^ = 0.25); hence, the SOV status cannot only be explained by its genetics [7,23]. Lastly, it is noteworthy that queens originating from SOV− maternal lines may still produce SOV+ offspring, indicating that the trait is not strictly maternally inherited and may also be influenced by paternal genetics.

### 4.4. Improvement of the SOV Trait Due to Targeted Breeding and Targeted Mating

The potential influence of paternal genetics has been highlighted in recent studies. De la Mora et al. [21] reported that DWV levels could be affected by the drone lines with which the selected queens mated and De Iorio et al. [19] demonstrated that controlled maternal and paternal genetics, achieved through single-drone insemination (SDI), contribute to the enhancement of VSH. Evidently, this raises the question of the extent to which both targeted breeding through the maternal line and targeted mating through the paternal line have influenced the occurrence of the SOV+ trait. If selective breeding based solely on the maternal SOV status is insufficient to achieve consistent improvement, incorporating targeted mating may enhance the transmission of the SOV+ status. Here, targeted mating refers to the selective use of drones originating from SOV+ colonies for the fertilization of virgin queens. In this study, such matings were achieved either through instrumental insemination (II) using drones from SOV+ queens or by placing virgin queens at the land-mating station Kreverhille (RKH), where only colonies confirmed as SOV+ within the selection program are maintained. Unfortunately, the available dataset remains limited due to the sparse occurrences of these targeted matings over the years. Between 2017 and 2021, II-targeted matings were performed, and from 2022 onwards, the use of the RKH mating station provided an additional, more natural route for targeted mating. However, only ≈41% of all II (and RKH) matings were with SOV+ drone lines (i.e., targeted matings). Consequently, firm conclusions regarding the additive effect of targeted mating remain difficult.

The combination of targeted breeding and targeted mating is expected to promote the inheritance and persistence of the SOV+ trait in the next generation. This combination occurred in only 46 cases over eight years of SOV sampling. Nevertheless, of all queens bred from an SOV+ maternal line, which was also mated with an SOV+ drone line (i.e., paternal line), ≈83% had an SOV+ status. Therefore, our findings suggest promising potential for reinforcing the SOV+ trait through combined maternal and paternal selection; however, the limited number of cases precludes drawing definitive statistical conclusions.

To strengthen future selection outcomes, stricter adherence to breeding guidelines may be warranted. Beekeepers participating in the program should ideally be obligated to breed exclusively from SOV+ queens to maintain selection intensity. Moreover, standardization of sampling across beekeepers and years would improve comparability, as variation in the number of queens tested per beekeeper and per year currently introduces additional noise into the dataset. Finally, it is important to note that SOV selection was not performed in isolation: participating colonies were also evaluated for Varroa resistance, and the SOV status served as a secondary selection criterion. This multifactorial approach may have diluted the direct selection pressure on viral resistance traits, underscoring the need for a more integrated and consistent breeding strategy in future programs.

### 4.5. Description of the SOV− Status

Examining the SOV− status provides additional insight into our efforts to improve the viral dynamics underlying the observed changes in SOV prevalence. The SOV– status indicates the presence of at least one virus in an egg sample. Across five sampling years (2020–2024), both the detection probability and the viral loads of the honey bee viruses—ABPV, BQCV, DWV, and SBV—from SOV− queens were evaluated. An overall decline in infection prevalence and intensity was observed for BQCV, DWV and SBV, suggesting an improvement in the virological health of the SOV-tested queen population over time. However, ABPV did not follow the same trends.

Our analysis suggests a shift in ABPV dynamics between 2020 and 2024, with a higher probability of detection of ABPV, but with lower viral loads. This pattern may indicate a change from rare, high-viral load infections to more widespread but lower-level circulation of the virus. Such patterns are consistent with viral adaptation toward reduced virulence or with enhanced antiviral defense mechanisms in the host population [24,25]. Alternatively, these results could reflect lower-intensity infections that are more readily detected by sensitive methods but are biologically less severe. In general, the virulence of the different ABPV strains remains poorly understood [16]. However, ABPV-infected queens were detected in only 2020 and 2024, while all queens were ABPV-free in the remaining three sampling years (2021–2023). Consequently, the number of positive samples was limited (*n* = 14), and our findings must be interpreted with caution. The observed increase in detection probability and decrease in viral load values could represent a true epidemiological trend but may also reflect stochastic variation or differences in sampling effort.

BQCV, DWV, and SBV each showed both declining detection probabilities and lower viral load values over time, suggesting a real reduction in infection pressure in Flanders. Whereas in the initial screenings (2012 and 2014) in Flanders, the most abundantly detected viruses were DWV and SBV, while BQCV seemed to emerge, at that time, albeit at moderate to low incidences [6]. Several hypotheses may explain these decreasing trends: improved Varroa control or management practices, colony-level resistance or tolerance mechanisms, or competitive interactions with other viruses. The occurrence of viruses is closely related to the presence of the Varroa mite, as the parasite vectors virus particles and triggers virus replication in honey bees or suppresses the immune response of bees as a consequence of Varroa parasitism or in an opportunistic way, benefiting from the general weakening of honey bees caused by the mite [5,26,27,28]. Likely, the decreasing trend of these viruses is related to the continuous efforts in Varroa control. This can be achieved through beekeeping practices (e.g., conventional chemical treatments), which is plausible for the beekeepers participating in our breeding program. Furthermore, it can be achieved through honey bees acquiring resistance or tolerance mechanisms. In terms of the latter, the current selection program also aims to breed resilient bees against Varroa. These decreasing trends also align with previous studies reporting that selective breeding towards Varroa resistance can lead to reduced virus prevalence and replication rates in managed honey bee populations [21,25,29]. To further substantiate this hypothesis, additional research should simultaneously monitor virus prevalence, Varroa infestation levels, and the presence of heritable Varroa-resistant traits (e.g., hygienic behavior, MNR, etc.) to disentangle their combined effects on colony health and viral dynamics.

When considering other potential explanatory variables for the observed trends, neither the type of mating (targeted vs. non-targeted) nor the honey bee subspecies showed a significant independent effect on viral load for any of the examined viruses. This could indicate that the observed reduction in virus infections is likely not driven by mating type alone or by genetic background at the subspecies level, but rather by selection pressure applied through the SOV-based breeding program itself. Nonetheless, this does not exclude the possibility that targeted mating may support the selection program when applied together with targeted breeding, but on its own, it does not influence viral load.

In the early years of sampling (2015–2018), queens occasionally carried multiple viruses, whereas since 2019, no samples have contained more than two viruses. This declining presence of multiple-virus infections further supports the positive effect of selective breeding towards SOV+ queens. Another hypothesis, here, could be that there are competitive interactions between viruses [30,31]. However, our data do not support this interpretation, as the presence of one particular virus does not take precedence over the presence of the other viruses over time. This pattern rather reflects improved overall viral resistance in the selected stock, although methodological factors—such as changes in detection sensitivity and differences in sample size—cannot be fully excluded.

## 5. Conclusions

Overall, our findings demonstrate that incorporating the SOV trait as a selection criterion can contribute to measurable progress in a honey bee selection breeding program. The occurrence of the SOV+ status significantly increased over time, and targeted breeding further enhanced this trend. Moreover, due to the observed decline in virus prevalence, infection intensity, and multi-virus co-infections among SOV− queens, we suggest that the selection strategy has contributed to an overall improvement in viral resilience in managed honey bee colonies, although direct fitness effects such as overwintering survival were not statistically resolved in this study. This study also emphasizes the potential to further improve the occurrence of the SOV trait by integrating targeted mating into selection breeding programs. Continued monitoring, with balanced sampling across years, standardized molecular diagnostics and stricter adherence to targeted breeding guidelines combined with targeted mating, will be essential to confirm whether progress towards virus resistance can be sustained and further elevated.

## Figures and Tables

**Table 1:** Overview of different observed and measured variables per sampling year (2015–2024).

	Sampling Year	2015	2016	2017	2018	2019	2020	2021	2022	2023	2024
***Participation in SOV testing***											
**A**	No. tested queens			184	178	218	279	379	306	312	289
**B**	No. SOV-tested queens	83	195	176	170	195	187	274	216	205	220
**C**	Observed percentage of SOV-tested queens (%)			95.65	95.51	89.45	67.03	72.30	70.59	65.71	76.12
***Improvement of the SOV+ status***											
**D**	No. SOV+ queens	47	129	73	110	173	88	195	206	179	188
**E**	Observed percentage of SOV+ queens (%)	56.63	66.15	41.48	64.71	87.72	47.06	71.17	95.37	87.32	85.45
***Improvement of the SOV+ status due to targeted breeding***											
**F**	No. SOV+ queens			20	58	56	31	83	83	78	75
**G**	Observed percentage of SOV+ queens (%)			27.40	52.73	32.37	35.23	42.56	40.29	43.58	39.89
***Improvement of the SOV+ status due to targeted breeding and targeted mating***											
**H**	No. targeted matings	56									
**I**	Observed percentage of targeted mating w.r.t. all matings via II/RKH (%)	40.58									
**J**	No. targeted breeding’s	613									
**K**	Observed percentage of targeted breeding w.r.t. all SOV-tested maternal lines (%)	85.61									
**L**	No. queens due to targeted breeding & targeted mating	46									
**M**	No. SOV+ queens due to targeted breeding and targeted mating	38									
**N**	Observed percentage of SOV+ queens due to targeted breeding and targeted mating w.r.t. all SOV-tested queens due to targeted breeding and targeted mating (%)	82.61									

**Table 2:** Overview of the number and observed percentages of SOV+ queens for every generation.

		Generation 1	Generation 2	Generation 3	Generation 4	Generation 5
***Improvement of the SOV+ status***						
**A**	No. SOV+ queens	85	284	133	83	53
**B**	Observed percentage of SOV+ queens (%)	73.91	75.53	77.33	82.18	81.54

**Table 3:** Overview of virus-infected queen numbers across sampling years (2020–2024). ABPV = acute bee paralysis virus, BQCV = black queen cell virus, DWV = deformed wing virus and SBV = sacbrood virus. ‘–’ indicates that no virus-infected queens were detected in that sampling year.

Sampling Year		2020	2021	2022	2023	2024
**ABPV**						
**A**	No. virus-infected queens	4	–	–	–	10
**BQCV**						
**B**	No. virus-infected queens	18	63	1	19	10
**DWV**						
**C**	No. virus-infected queens	72	20	2	6	–
**SBV**						
**D**	No. virus-infected queens	22	–	8	1	13

## Data Availability

The data presented in this study are available on request from the corresponding author. The data are not publicly available due to privacy restrictions.

## Supplementary Materials

The following supporting information can be downloaded at https://www.mdpi.com/article/10.3390/insects17020137/s1: Figure S1: Schematic overview of the workflow of the selective breeding program at Honeybee Valley; Figure S2: Schematic overview of the timetable of SOV testing; Table S1: Representativeness of the sample collection over sampling years; Table S2. Concordance testing between RT-PCR and RT-qPCR with DWV-positive results; Table S3: Sequence of the used primers in RT-PCR/qRT-PCR.

## Abbreviations

The following abbreviations are used in this manuscript:

SOV	Suppressed *in ovo* virus infection
DWV	Deformed wing virus
BQCV	Black queen cell virus
ABPV	Acute bee paralysis virus
CBPV	Chronic bee paralysis virus
SBV	Linear dichroism
RNA	Ribonucleic acid
cDNA	Copy DNA or complementary DNA (DNA = Deoxyribonucleic acid)
RT-PCR	Reverse Transcription Polymerase Chain Reaction
RT-qPCR	Reverse Transcription quantitative Polymerase Chain Reaction
GLMM	Generalized linear mixed model
GLM	Generalized linear model
AIC	Akaike Information Criterion
SE	Standard error
CI	Confidence interval
II	Instrumental insemination
RKH	Mating station Kreverhille
