# Supplementary material for: Evaluation of 10-Year Selection for Virus Resistance in a Mass Breeding Program
Source: Insects. 2026 Jan 24;17(2):137. doi: 10.3390/insects17020137 (PMC12940707; doi:10.3390/insects17020137)
Supplement: Supplementary file 1 [file insects-17-00137-s001.zip › insects-4082905-supplementary.pdf]

Supplementary Information

Evaluation of 10-Year Selection for Virus Resistance in a Mass Breeding Program

Emma Bossuyt [1,*], Marleen Brunain [2], Lina De Smet [2], Ellen Danneels [1], Dirk C. de Graaf [1,2]

Supplementary Figure S1. Schematic overview of the workflow of the selective breeding program at Honeybee Valley.

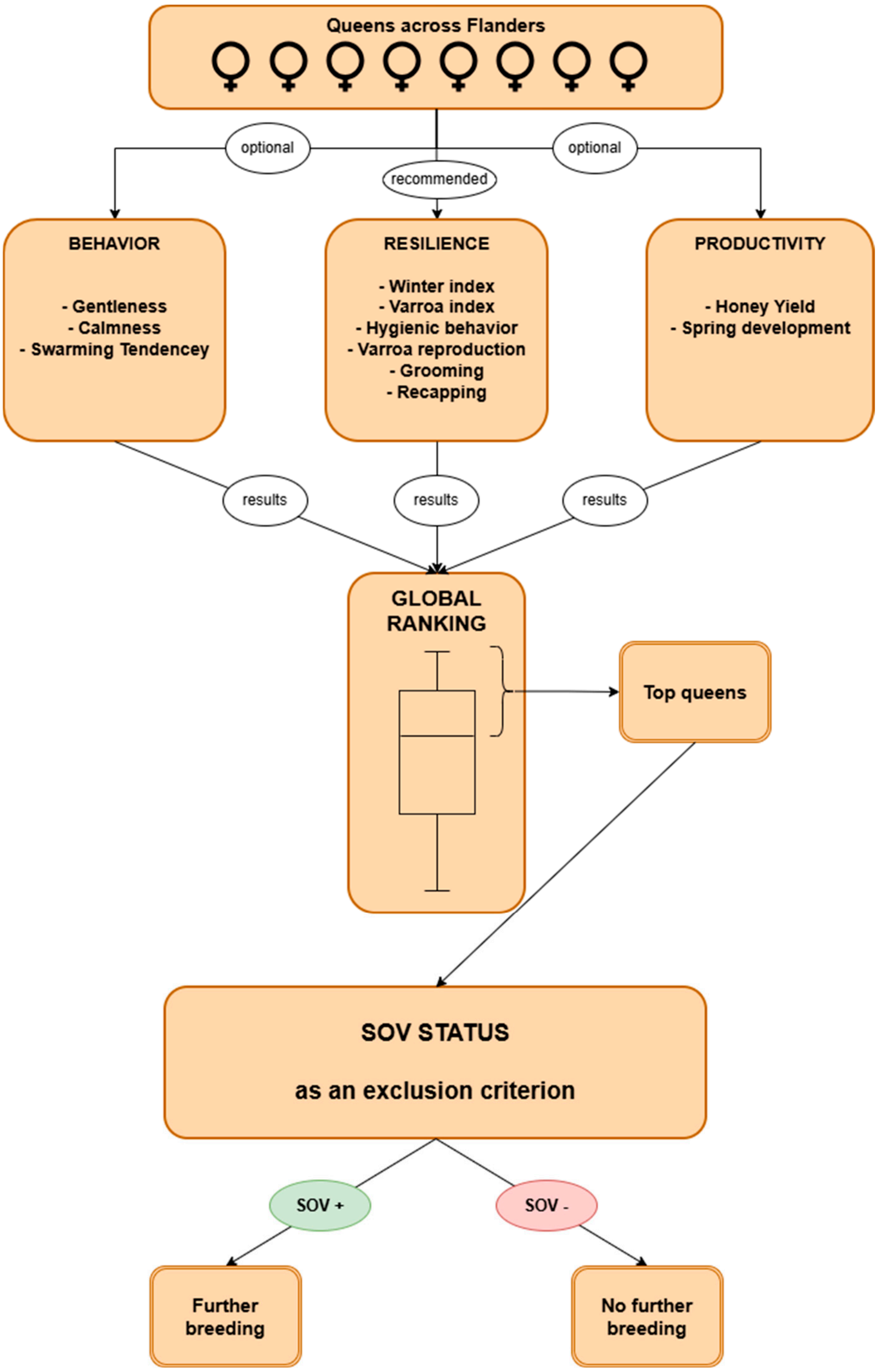

Supplementary Figure S2. Schematic overview of the timetable of SOV testing.

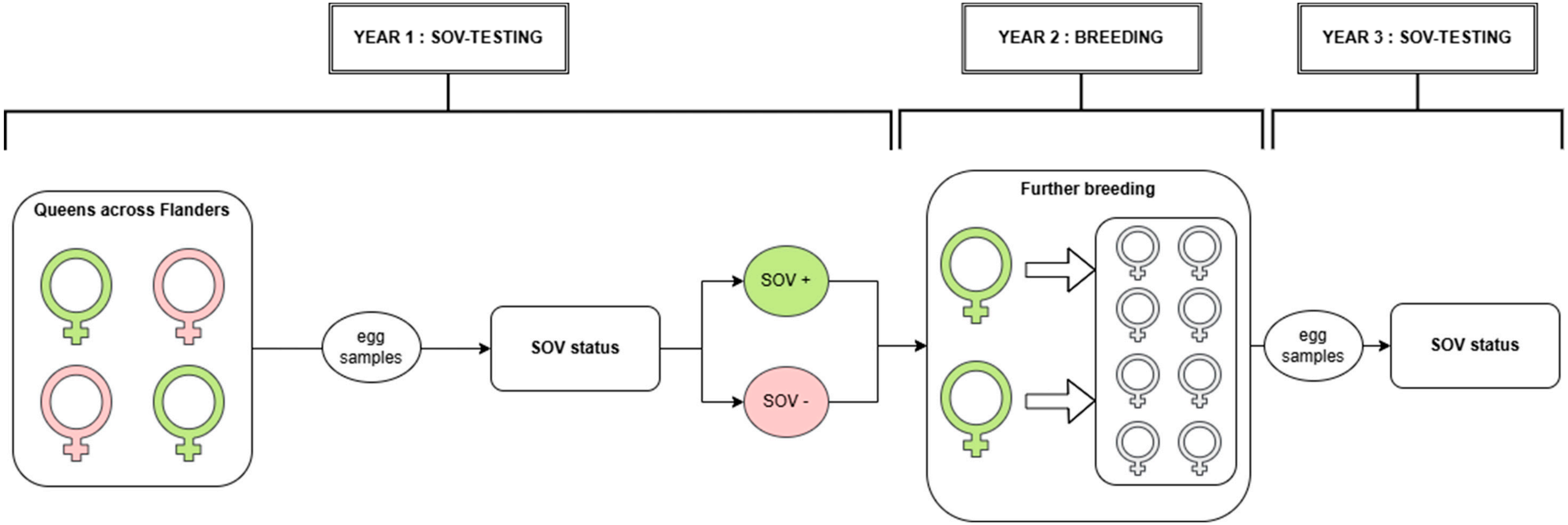

Supplementary Table S1. Representativeness of the sample collection over sampling years.

**Sampling Year**	**2015**	**2016**	**2017**	**2018**	**2019**	**2020**	**2021**	**2022**	**2023**	**2024**
No. SOV-tested queens	83	195	176	170	195	187	274	216	205	220
No. participating beekeepers	15	40	33	25	27	32	40	37	36	37
Arithmetic mean no. SOV-tested queens per beekeeper	5.53	4.88	5.33	6.80	7.22	5.84	6.85	5.84	5.69	5.95

Supplementary Table S2. Concordance testing between RT-PCR and RT-qPCR with DWV-positive results.

	Viral load per egg determined by RT-qPCR	Positive samples with RT-PCR/total samples tested
1	10E12	1/1
2	10E11	1/1
3	10E10	3/3
4	10E9	2/2
5	10E8	2/2
6	10E7	3/3
7	10E6	2/3
8	10E5	3/4
9	10E4	3/4
10	10E3	1/2

Supplementary Table S3. Sequence of the used primers in RT-PCR/qRT-PCR.

Name	Sequence
ABPV-F	TCA TAC CTG CCG ATC AAG
ABPV-R	CTG AAT AAT ACT GTG CGT ATC
BQCV-F	AGT GGC GGA GAT GTA TGC
BQCV-R	GGA GGT GAA GTG GCT ATA TC
DWV-F	TTC ATT AAA GCC ACC TGG AAC ATC
DWV-R	TTT CCT CAT TAA CTG TGT CGT TGA
SBV-F	TTG GAA CTA CGC ATT CTC TG
SBV-R	GCT CTA ACC TCG CAT CAA C
DWV_ F8688	GGT AAG CGA TGG TTG TTT G
DVW_ B8794 R	CCG TGA ATA TAG TGT GAG G
Am_actine_F	TGC CAA CAC TGT CCT TTC TG
Am_actine_R	AGA ATT GAC CCA CCA ATC CA
ß-actine_F	CGT GCC GAT AGT ATT CTT G
ß-actine_R	CTT GTC ACC AAC ATA GG
